# Nightmares in Swiss elite athletes: Associated factors

**DOI:** 10.1111/jsr.14283

**Published:** 2024-07-01

**Authors:** Michael Schredl, Albrecht Vorster, Michael J. Schmid, Daniel Erlacher

**Affiliations:** ^1^ Central Institute of Mental Health, Medical Faculty Mannheim Heidelberg University Mannheim Germany; ^2^ Swiss Sleep House Bern, Department of Neurology University Hospital Bern Bern Switzerland; ^3^ Institute of Sport Science University of Bern Bern Switzerland

**Keywords:** elite athletes, gender differences, nightmares, stress

## Abstract

Nightmares, defined as extremely dysphoric dreams, can cause significant distress in everyday life if they occur frequently. Their aetiology is based on a disposition‐stress model. As elite athletes often experience high stress levels, the present study investigated factors that might be associated with nightmare frequency in a large cohort of 2297 Swiss elite athletes (1066 women, 1231 men) with a mean age of 22.05 ± 7.53 years. In total, about 6% of the athletes reported frequent nightmares (once a week or more often). We found that well‐established factors like female gender and general stress levels were related to nightmare frequency. To a smaller extent, the number of training hours, lost training days due to illness, and having early training sessions were also associated with nightmare frequency. Sport discipline was not related to nightmare frequency. An unexpected finding was the association between late alcohol intake 4 hr prior to bedtime and nightmare frequency. Our findings support the idea that stress related to practicing sports might affect nightmare frequency. Future research should study whether inventions designed for athletes suffering from frequent nightmares are beneficial for them and might even improve their athletic performance.

## 1 INTRODUCTION

Nightmares are defined as extended, extremely dysphoric and well‐remembered dreams that usually involve threats to survival, security or physical integrity (American Academy of Sleep Medicine, 2014). Typical themes are physical aggression, interpersonal conflicts, failure/helplessness, accidents, being chased, health‐related issues and death (Robert & Zadra, 2014; Schredl & Göritz, 2018). Whereas almost everyone has experienced occasional nightmares during childhood, adolescence or young adulthood (Schredl et al., 2014), 2%–6% of adults report frequent nightmares occurring once a week or more often (Levin & Nielsen, 2007). These persons usually often show significant distress in their waking life and might be diagnosed with a nightmare disorder (American Academy of Sleep Medicine, 2014). Nightmare aetiology is described by a disposition‐stress model (Schredl, 2023), including genetic factors, personality dimensions (neuroticism, “thin boundaries”) and stressors. Given the research findings showing that elite athletes often face high stress levels (McLoughlin et al., 2021; Ward et al., 2023), research has explored whether this group often experience nightmares. On the other hand, personality factors seem not to point to a heightened vulnerability to nightmares as elite athletes reported higher emotional stability and higher conscientiousness (Steca et al., 2018).

The most extensive study to date, conducted by Erlacher et al. (2011), surveyed 840 German athletes and found that 5.36% experienced frequent nightmares, occurring at least once per week. Sport‐related variables like sport type (individual versus group sports) and the number of training hours per week did not correlate with nightmare frequency; however, women tended to report nightmares more often than men (Erlacher et al., 2011). Similarly, Gan et al. (2022) found no an association between sport‐related variables such as sport type, skill level, training years and nightmares; only self‐rated anxiety levels in waking were linked to nightmare frequency. This is comparable to findings regarding nightmares in non‐athlete samples reporting that gender (Schredl & Reinhard, 2011) and neuroticism—including anxiety—(Carr et al., 2022; Gessert & Schredl, 2023; Schredl & Göritz, 2021) are related to nightmare frequency. Given the small number of studies with heterogeneous samples regarding the athletes' skill levels, the question as to whether sport‐related variables might be associated with nightmare frequency in athletes is still open for discussion.

The present study addressed this open question. We expected that sport‐related variables that are specifically associated with stress like performance levels, sport‐related injuries, number of days with competition, and training hours correlate with nightmare frequency—in addition to well‐established factors like female gender and general stress levels.

## 2 METHODS

### 2.1 Participants

Overall, 2297 participants (1066 women, 1231 men) completed the online survey. The mean age was 22.05 ± 7.53 years (range: 16–68 years, one missing value). The athletes practiced and competed in different sports disciplines: *N* = 168 artistic composition sports (e.g. artistic gymnastics, rhythmic gymnastics, figure skating, freestyle skiing); *N* = 958 cgs sports (performance is measured in centimetres, grams or seconds, e.g. alpine skiing, cycling, rowing, swimming, track and field); *N* = 137 combat sports (e.g. boxing, judo, wrestling, fencing); *N* = 874 game sports (e.g. basketball, football, floorball, tennis); *N* = 39 air and motor sports (e.g. sport flying); *N* = 84 (equestrian sports). The rest of the group consisted of various disciplines including the parasports and wheelchair sports. For four participants, no information about their sport discipline was available. A total of 300 athletes belong to the world class (top 10), 634 to the extended world class (top 10–50) and 1253 to the national top class (top 10).

### 2.2 Research instruments

For assessing nightmare frequency, an eight‐point rating scale (“How often have you experienced nightmares recently [in the past several months]?”) ranging from 0 = never, 1 = less than once a year, 2 = about once a year, 3 = about two–four times a year, 4 = about once a month, 5 = two–three times a month, 6 = about once a week, 7 = several times a week was used (Schredl et al., 2014). The scale included the following definition: “Nightmares are dreams with strong negative emotions that result in awakening from the dream. The dream plot can be recalled very vividly upon awakening”. The retest reliability of this scale over a 2‐week period was high: *r* = 0.765 (Schredl et al., 2014).

The general stress level over the last months was measured by a five‐point scale: 1 = very low, 2 = low, 3 = moderate, 4 = high and 5 = very high. The participants were asked to take areas like study time, profession and leisure time into account. For measuring chronotype, one item of the German version of the Morning–Evening Questionnaire (MEQ) was used (Griefahn et al., 2001): the participants could choose between the following four options: I consider myself to be “definitely a morning type”, “rather a morning type than an evening type”, “rather an evening type than a morning type”, or “definitely an evening type”. The retest reliability over a 6‐year period of this item was high: *r* = 0.741 (Staller et al., 2023). In addition, the typical sleep duration was assessed in hours.

Several questions were related to training and competition in the past year: (1) weekly training hours and the number of competition; (2) injuries that required medical assistance (response options: none, one, and two or more); (3) number of missed training days due to illness (response options: 0 days missed, 1–10 days missed, and more than 10 days missed); (4) frequency of training and competition prior to 07:00 hours or after 19:00 hours using five response options (i.e. “More than 5 times per week”, “3–5 times per week”, “1–2 times per week”, “1–3 times per month”, and “Never or rarely”).

Moreover, the athletes were also asked about their nicotine (snus, cigarettes, nicotine gums, nicotine patches), caffeine/energy drink, and alcohol consumption after 18:00 hours (for alcohol, the interval was 4 hr before bedtime) using again five response options (i.e. “More than 5 times per week”, “3–5 times per week”, “1–2 times per week”, “1–3 times per month”, and “Never or rarely”).

Finally, the athletes' gender, age, type of sport, weight and height were also elicited.

### 2.3 Procedure

The study was carried out in collaboration with Swiss Olympic, the National Olympic Committee and the umbrella organization for organized Swiss sport under private law. The invitation to participate was sent by e‐mail to all athletes (16 years and older) in Switzerland who are a member of a national squad at a junior or elite level. Of these 5188 athletes, 2297 athletes completed the survey (response rate: 44.28%). The online survey (programmed on LimeSurvey, version 2.50) was sent out on 17 November 2023 and lasted until 10 December 2023. The participants came from 100 different sports, including alpine skiing, athletics, football, artistic gymnastics, ice hockey, rowing, swimming, tennis and other disciplines. For the purpose of this analysis, these disciplines were categorized into several groups (see Section 2.1).

The study protocol was approved by the ethics committee of the Faculty of Human Sciences of the University of Bern, and was conducted in accordance with the Declaration of Helsinki.

Statistical procedures were carried out using SAS software package for Windows 9.4 (SAS Institute, Cary, North Carolina, USA). As the nightmare scale is ordinal, ordinal regressions were computed.

## 3 RESULTS

The distribution of the nightmare frequency scale is shown in Table 1; about 6% of the athletes reported having nightmares once a week or more often, whereas most reported infrequent or no nightmares. The general stress level over the last months was distributed as follows: very low (*N* = 34; 1.48%); low (*N* = 378; 16.46%); moderate (*N* = 1083; 47.15%); high (*N* = 703; 30.61%); very high (*N* = 99; 4.31%). The body mass index mean is within the normal range (Table 2). The total sleep duration was about 7.5 hr on average. The athletes trained on average about 15 hr per week, and participated in competitions almost 30 days during the past year. One injury that required medical assistance during the past year was reported by 802 athletes (34.92%), whereas 497 athletes (21.64%) reported two or more injuries. No injuries were reported by 998 athletes (43.45%). In total, 438 athletes (19.07%) missed no training days due to illness; 1275 athletes (55.51%) missed 1–10 days; and 584 athletes (25.42%) missed more than 10 days. Regular early morning training/competition (once a week or more often) was reported by about 9% of the athletes, whereas almost 50% reported training/competitions after 19:00 hours in the evening. Consuming caffeine/energy drinks more than once a week after 18:00 hours was reported by about 11% (Table 3), the percentages of nicotine consumers (about 6%) and regular alcohol consumption (about 5%) were low. Overall, 219 athletes (9.53%) stated to be “definitely morning type”, 724 athletes (31.52%) “rather a morning type than an evening type”, 934 athletes (40.66%) “rather an evening type than a morning type”, and 420 athletes (18.28%) “definitely an evening type”.

**TABLE 1:** Nightmare frequency distribution (*N* = 2297).

Category	*N*	Percentage
Several times a week	30	1.31
About once a week	115	5.01
Two or three times a month	227	9.88
About once a month	374	16.28
About two or four times a year	633	27.56
About once a year	293	12.76
Less than once a year	202	8.79
Never	423	18.42

**TABLE 2:** Means and standard deviations.

Category	Mean ± SD
Training hours per week (*N* = 2297)	15.04 ± 7.01
Competition days (last year) (*N* = 2270)	28.42 ± 18.14
Body mass index (*N* = 2294)	22.56 ± 2.63
Total sleep time in hours (*N* = 2297)	7.60 ± 0.86

**TABLE 3:** Training/competition timing and consumption in the evening (*N* = 2297).

	Training or competition		Consumption		
Category	Prior to 07:00 hours	After 19:00 hours	Caffeine/energy drinks after 18:00 hours	Nicotine after 18:00 hours	Alcohol after 4 hr prior to bedtime
More than 5 times per week	13 (0.57%)	158 (6.88%)	25 (1.09%)	92 (4.04%)	2 (0.09%)
3–5 times per week	53 (2.31%)	543 (23.64%)	68 (2.96%)	19 (0.83%)	12 (0.52%)
1–2 times per week	142 (6.18%)	418 (18.20%)	161 (7.01%)	22 (0.96%)	95 (4.14%)
1–3 times per month	237 (10.32%)	417 (18.15%)	325 (14.15%)	49 (2.13%)	531 (23.12%)
Never or rarely	1852 (80.63%)	761 (33.13%)	1718 (74.79%)	2115 (92.08%)	1637 (72.14%)

The ordinal regression for nightmare frequency with all variables entered simultaneously showed that there are three main factors associated with nightmare frequency: female gender; general stress levels; and alcohol consumption 4 hr before bedtime (Table 4). The number of training hours, lost training days to illness, and having early training/competitions were also significantly associated with nightmare frequency, albeit the effect sizes were very small. Other variables like total sleep time, chronotype, body mass index, caffeine/nicotine consumption, age, performance levels, having injuries, having late training sessions or competition were not related to nightmare frequency. An additional analysis including the sport groups (artistic composition sports, cgs sports, combat sports, game sports, air and motor sports, and equestrian sports) in addition to the variables shown in Table 4 yielded no significant differences regarding to the type of sport practiced (results not presented).

**TABLE 4:** Ordinal regression analysis for nightmare frequency (*N* = 2160).

Variable	Coefficient	*χ* ^2^	*p*	Effect size
Age	−0.0324	1.8	0.1804	0.058
Gender (1 = f, 0 = m)	0.1707	56.6	< 0.0001^a^	0.328
Stress level	0.1473	42.7	< 0.0001^a^	0.284
Performance level	−0.0234	1.1	0.8536^a^	0.045
Injuries	0.0306	1.9	0.0841^a^	0.059
Illnesses	0.0446	4.0	0.0228^a^	0.086
Number of training hours per week	0.0461	4.1	0.0220^a^	0.087
Competitions	−0.0430	0.9	0.1674^a^	0.041
Caffeine after 18:00 hours	−0.0056	0.1	0.7874	0.014
Nicotine after 18:00 hours	0.0036	0.0	0.8724	0.008
Alcohol intake 4 hr prior to bedtime	0.0922	16.7	< 0.0001	0.177
Chronotype	−0.0167	0.6	0.4503	0.033
Body mass index	−0.0249	1.2	0.2764	0.047
Total sleep time	0.0143	0.4	0.5400	0.072
Early training	0.0445	4.3	0.0195^a^	0.089
Late training	0.0244	1.1	0.1430	0.045

^a^ One‐tailed, adj. *R* ^2^ = 0.0412 for the regression model.

## 4 DISCUSSION

Overall, about 6% of the athletes reported nightmares once a week or more often. As expected, well‐established factors like female gender and general stress levels were related to nightmare frequency. We found small but significant associations between nightmare frequency and illness, training hours, and having early training sessions. This supports the idea that stress related to practicing sports on an elite level might also affect nightmare frequency. Moreover, we observed a significant association between late alcohol intake and nightmare frequency. However, the sport discipline was not related to the nightmare frequency.

From a methodological viewpoint, it is important to take the response rate of 44.28% into consideration, especially if evaluating the percentage of persons with frequent nightmares. As the study was about sleep and sports, one might speculate whether athletes with sleep problems or nightmares were more willing to participate in the study. However, the conservative estimate would be that at least 3% of the elite athletes in Switzerland experience nightmares once per week or more often. As this frequency might mark the presence of a nightmare disorder (Schredl, 2023), it would be very interesting to conduct further studies regarding the question of whether athletes might benefit from nightmare treatment, for example, Imagery Rehearsal Therapy (Krakow & Zadra, 2010). On the other hand, the response rate of 44.28% should have no or only minor effects on the inter‐relationships between nightmare frequency and the various predictors as the variance of the scales is not restricted, that is, the whole range, for example, from no nightmares to frequent nightmares is found in the present sample.

The percentage of athletes reporting frequent nightmares in the present study is comparable to the 5.36% of German athletes reported by Erlacher et al. (2011). Whether this percentage is increased compared with the general population (range of 2%–6%; Levin & Nielsen, 2007) is an open question. Even though the percentage of 6.3% of athletes with frequent nightmares is slightly higher compared with percentages in representative samples (Schredl, 2010, 2013), one has to keep in mind that nightmare frequency decreases with age in adults (Schredl et al., 2014). Thus, higher percentages would be expected in samples of young adults like the present sample of Swiss elite athletes. Given the small but significant associations with sport‐related variables (see below), one might speculate that athletes experience nightmares slightly more often than non‐athletes.

Based on a meta‐analysis of 42 studies (Schredl & Reinhard, 2011), the gender difference regarding nightmare frequency in young adults (general population) was estimated at an effect size of 0.263. Finding a gender effect in a similar range (*d* = 0.328) supports the validity of the present study, especially as a gender difference in nightmare frequency was also reported in a previous sample of athletes (Erlacher et al., 2011). Similarly, the effect size of the association between general stress levels and nightmare frequency is comparable to previous studies (Carr et al., 2022; Gessert & Schredl, 2023; Schredl & Göritz, 2021); again, supporting the validity of the present findings.

Several sport‐related variables like performance levels, competition days and late training were not significantly related to nightmare frequency. Although the effect sizes were relatively small, other sport‐related variables like suffering from injuries (marginally significant), being ill that affects training, number of training hours, and having early training were related to nightmare frequency. Thus, sport‐related stress can result in more nightmares. It would be very interesting to expand the present study and include items that target the subjective sport‐related stress in addition to the general stress levels that were measured in the present study. It could be argued that the subjective side of experiencing injuries (e.g. worries about the future of one's career) or the stress associated with important competitions (especially if they did not go well) may be more closely linked to nightmares than the crude measure whether or not such an event occurred. In addition, the question whether early training is more stressful to athletes compared with late training could be pursued further.

An interesting finding was the association between alcohol consumption 4 hr before bedtime and nightmare frequency, as this has not been reported in the literature. Only one large‐scaled study in Japanese adolescents reported that overall alcohol intake was related to nightmare frequency (Munezawa et al., 2011). From a physiological viewpoint, this makes sense as alcohol consumption prior to bedtime suppresses rapid eye movement (REM) sleep in the first part of the night, and thus results in a REM rebound in the second part of the night (McCullar et al., 2024). This more intense REM sleep might be accompanied by more intense dreaming (Fiss et al., 1974). Unfortunately, systematic experimental studies on the effect of alcohol intake prior to bedtime and nightmare occurrence are lacking. Another line of thinking might be that alcohol is used—by a very small percentage of athletes in the present sample—to regulate stress‐related tension, that is, athletes regularly drinking alcohol in the evening might be more stressed and, therefore, experience more nightmares. However, this explanation seems more unlikely, as the effect of general stress levels was statistically controlled for, that is, the alcohol effect was independent of the stress effect.

To summarize, the present study demonstrated that sport‐related variables had a small effect on nightmare frequency, and thus frequent nightmares might be a problem for some athletes. This is especially valid as nightmares are often associated with poor sleep quality (Delage et al., 2024) and might be partly responsible for the relative high percentage of athletes with sleep problems (Charest & Grandner, 2022). It would be very interesting to carry out invention studies with athletes suffering from frequent nightmares, for example, using Imagery Rehearsal Therapy (Krakow & Zadra, 2010), as such interventions might even improve their performance.

## AUTHOR CONTRIBUTIONS

**Michael Schredl:** Formal analysis; writing – original draft; writing – review and editing; conceptualization. **Albrecht Vorster:** Conceptualization; methodology; investigation; data curation; project administration; writing – review and editing. **Michael J. Schmid:** Conceptualization; methodology; software; data curation; project administration; writing – review and editing. **Daniel Erlacher:** Conceptualization; methodology; project administration; writing – review and editing.

## FUNDING INFORMATION

No funding.

## CONFLICT OF INTEREST STATEMENT

The authors have nothing to declare.

## Data Availability

The data that support the findings of this study are available from the corresponding author upon reasonable request.
